# Anti-Cripto Mab inhibit tumour growth and overcome MDR in a human leukaemia MDR cell line by inhibition of Akt and activation of JNK/SAPK and bad death pathways

**DOI:** 10.1038/sj.bjc.6603641

**Published:** 2007-03-06

**Authors:** X F Hu, J Li, E Yang, S Vandervalk, P X Xing

**Affiliations:** 1 Cancer Immunotherapy Laboratory, Burnet Institute Incorporating Austin Research Institute, Heidelberg, Australia

**Keywords:** Cripto Mab, MDR, P-glycoprotein, apoptosis, signalling transduction pathways

## Abstract

Doxorubicin (DOX) selection of CCRF-CEM leukaemia cell line resulted in multidrug resistance (MDR) CEM/A7R cell line, which overexpresses MDR, 1 coded P-glycoprotein (Pgp). Here, we report for the first time that oncoprotein Cripto, a founding member of epidermal growth factor-Cripto-FRL, 1-Criptic family is overexpressed in the CEM/A7R cells, and anti-Cripto monoclonal antibodies (Mab) inhibited CEM/A7R cell growth both *in vitro* and in an established xenograft tumour in severe combined immunodeficiency mice. Cripto Mab synergistically enhanced sensitivity of the MDR cells to Pgp substrates epirubicin (EPI), daunorubicin (DAU) and non-Pgp substrates nucleoside analogue cytosine arabinoside (AraC). In particular, the combination of anti-Cripto Mab at less than 50% of inhibition concentrations with noncytotoxic concentrations of EPI or DAU inhibited more than 90% of CEM/A7R cell growth. Cripto Mab slightly inhibited Pgp expression, and had little effect on Pgp function, indicating that a mechanism independent of Pgp was involved in overcoming MDR. We demonstrated that anti-Cripto Mab-induced CEM/A7R cell apoptosis, which was associated with an enhanced activity of the c-Jun N-terminal kinase/stress-activated protein kinase and inhibition of Akt phosphorylation, resulting in an activation of mitochondrial apoptosis pathway as evidenced by dephosphorylation of Bad at Ser136, Bcl-2 at Ser70 and a cleaved caspase-9.

Despite advances made over the last 30 years, most acute leukaemia remains difficult to cure owing to the development of multidrug resistance (MDR) (Abd El-Ghaffar *et al*, 2006; Biscardi *et al*, 2006). The most well-characterised MDR gene product in relapsed acute leukaemia is P-glycoprotein (Pgp) encoded by MDR1 gene (Abd El-Ghaffar *et al*, 2006). P-glycoprotein, an ATP-binding protein, functions as an energy-dependent efflux pump resulting in a decreased accumulation of several structurally unrelated drugs including anthracyclines such as doxorubicin (DOX), epirubicin (EPI) and daunorubicin (DAU) and vinca alkaloids such as vinblastine and vincristine as well as epipodophyllotoxins leading to MDR phenotype (Hayes and Wolf, 1990). A number of reagents such as a calcium channel blocker verapamil (Vp), immunosuppressor cyclosporin A (CyA) and its nonimmunosuppressive derivative PSC833 overcame MDR by inhibition of Pgp function (Hu *et al*, 1990). However, the acquisition of Pgp-mediated MDR during chemotherapy remains poorly understood, and MDR cannot be fully overcome at this stage. A MDR variant CEM/A7R cell line, developed by DOX selection of T-cell lymphoblastic leukaemia CCRF-CEM cell line (Zalcberg *et al*, 1994), has been successfully used as a model to study the molecular mechanisms underlying regulation of drug resistance. A rapid upregulation of MDR1 gene in CEM/A7R cell line was induced by anthracyclines and its analogues (Hu *et al*, 1995, 1999b), and prevented by CyA and its analogue PSC 833 (Hu *et al*, 1996). These results represent the clinical development of Pgp-mediated drug resistance during chemotherapy (Hu *et al*, 1999a). Although Pgp expression plays a significant role in DOX-induced MDR phenotype, accumulated evidence indicated that additional molecular events besides MDR1 were involved (Watts *et al*, 2001). Recently oncoprotein Cripto, the founding member of the epidermal growth factor-Cripto-FRL1-Criptic (EGF-CFC) family, was also demonstrated to be an important factor in mediating drug resistance (Normanno *et al*, 2004). We have found that Cripto is upregulated in Pgp-positive CEM/A7R cells. Cripto was first identified and sequenced from a teratocarcinoma cDNA expressing library (Ciccodicola *et al*, 1989), and is overexpressed in most cancers, implicating its role in tumorigenesis and progression (Saloman *et al*, 2000).

Both Cripto and MDR1 have been demonstrated as new target genes of *β*-catenin (Yamada *et al*, 2000; Morkel *et al*, 2003), a key mediator of Wnt signalling pathway. Upregulation of MDR1 and accumulation of *β*-catenin have been observed in chemically induced rat and human liver adenomas and adenocarcinomas (Yamada *et al*, 1999). It is more likely that chemically induced intracellular accumulation of *β*-catenin leads to its nuclear translocation and binding to T-cell factor and lymphoid enhancer factor transcription factors, which transactivated MDR1 and Cripto along with other Wnt target genes (Seidensticker and Behrens, 2000). This may explain why DOX selection of Pgp-mediated MDR phenotype in the CEM/A7R cells leads to simultaneous induction of Cripto expression.

Most chemotherapeutic agents induce tumour cells to undergo a final apoptosis pathway (Fisher, 1994). The defects in the apoptotic pathway represent an important mechanism for the development of chemo–resistance (Minn *et al*, 1995). Cripto possesses an N-terminal signal peptide, a unique EGF-like motif, a cysteine-rich CFC domain and a short hydrophobic carboxy terminus, which is essential for membrane anchorage by a glycosylphosphatidylinositol (GPI) moiety (Saloman *et al*, 2000). The EGF-like region of Cripto is involved in activation of c-Src (Bianco *et al*, 2003), ras/raf/MAPK (Kannan *et al*, 1997) and PI3k/Akt/GSK-3*β* (Ebert *et al*, 1999) mediated cell proliferation and cell survival signalling pathways. Constitutive activation of PI3K/Akt pathway promotes MDR phenotype in breast cancer (Clark *et al*, 2002), prostate cancer (Lee *et al*, 2004) and acute myeloid leukaemia (AML) (Grandage *et al*, 2005). These observations suggest that Cripto-activated PI3K/Akt pathway in CEM/A7R cells not only stimulates tumour growth but also induces MDR phenotype through interruption of apoptosis pathways activated by chemotherapeutics. In addition to drug efflux, Pgp overexpression in the CEM/A7R cells also renders cells a cross-resistance to caspase-dependent apoptosis stimulated by cytotoxics and apoptotic stimulus such as Fas ligand (Smyth *et al*, 1998; Johnstone *et al*, 1999). These results suggest that tumour cells may use both Cripto and Pgp protein to escape cell death, induced by chemotherapeutic drugs.

Cripto has been identified as a therapeutic target (Adkins *et al*, 2003; Xing *et al*, 2004), and the EGF-like region has been regarded as a suitable immunotherapeutic intervention point (Hu and Xing, 2005). Two monoclonal antibodies (Mab) C4 and C13 have been generated against a Cripto 17-mer peptide, corresponding to the ‘EGF-like’ motif of Cripto, and have shown effective inhibition of PI3K/Akt pathway and activation of c-Jun N-terminal kinase/stress-activated protein kinase (JNK/SAPK) pathways in colon cancer cells (Xing *et al*, 2004). In this paper, we are aiming to examine whether the Mab to Cripto could overcome the Pgp-mediated MDR and the signalling transduction pathways interrupted by the Mab. Here, we report that Mab C4 and C13 overcame MDR and inhibited tumour growth of the MDR CEM/A7R cells by inhibition of Akt and activation of JNK/SAPK and mitochondrial apoptotic pathway, demonstrating a new strategy for overcoming Pgp-mediated MDR.

## MATERIALS AND METHODS

### Cell lines and cytotoxic drugs

CEM/A7R is a variant of the CEM/A7 cell line, derived by stepwise selection in increasing concentrations of DOX from a drug-sensitive CCRF-CEM parental cell line (Zalcberg *et al*, 1994), originally developed from a patient with a T-cell lymphoblastic leukaemia (Foley *et al*, 1965). CEM/A7R line was established by growing the CEM/A7 cells in the absence of DOX for more than 2 years before subcloned in 96-well plates by a limited dilution technique (Hu *et al*, 1995). Daunorubicin, EPI and cytosine arabinoside (AraC) were obtained from Pharmacia & Upjohn Pty Limited (Perth, Australia). Verapamil was purchased from Knoll Pty Limited (NSW, Australia). Rh123 was obtained from Sigma (Australia). The CEM/A7R and CCRF-CEM cells were cultured in RPMI 1640 containing 10% heat-inactivated FCS at 37°C in a 5% CO_2_ humidified incubator.

### Cripto Mab

Production and characterisation of anti-Cripto EGF-like region Mab C4, C13 have been recently described (Xing *et al*, 2004). Briefly, a 17-mer peptide from the EGF-like region amino acids 97–113 (CPPSFYGRNCEHDVRKE) of Cripto was synthesised using an Applied Bio-systems Model 430A automated peptide synthesiser (Foster City, CA, USA) as immunogen. Murine myeloma NS1 cells were fused with spleen cells from Lewis female rats that were immunised three times. First immunization was injected intraperitoneally by 100 *μ*g Cripto 17-mer conjugated to keyhole limpet haemocyanin (KLH); and emulsified in complete Freund's adjuvant. After 4 weeks, a second injection of Cripto 17-mer-KLH with incomplete Freund's adjuvant, and 3 days later, a third injection was given without adjuvant. The supernatants of hybridomas were tested by enzyme-linked immunosorbent assay and immunohistochemistry for their activity and specificity. C4 and C13 Mab were further selected by cell growth assays, and purified from ascites by Sephacryl S300 HR gel filtration after 50% saturated ammonium sulphate precipitation. The subclasses of the Mab were determined using anti-rat immunoglobulin subclass antibodies (ICN, Irvine, CA, USA).

### Cell proliferation assays

[^3^H]-thymidine incorporation assay was performed as previously described (Hu *et al*, 1990). Briefly, 5 × 10^4^ well^−1^ of CEM/A7R or parental CCRF-CEM cells were cultured for 48 h at 37°C in 5% CO_2_ with varying concentrations of Cripto Mabs and chemotherapeutic agents DAU, EPI or AraC. The cells were pulsed with [^3^H]-thymidine (final concentration 1 *μ*ci ml^−1^) for 4 h and then harvested onto glass filter papers using an automated cell harvester and radioactivity was measured by TopCount (Perkin–Elmer, Boston, MA, USA). All assays were performed in triplicate and the results were expressed as percentage of [^3^H]-thymidine incorporation in the treated group to control (medium only) (Xing *et al*, 2004). Inhibition of cell growth by Mab equals the percentage of thymidine incorporation of control minus that of treated samples. The results obtained from the assay are comparable from cell growth inhibition assay by using the trypan blue dye exclusion method (Hu *et al*, 1990; Xing *et al*, 2004).

### Tumour eradication induced by Mab in SCID mice

Severe combined immuno deficiency (SCID) mice (female, 10–12 weeks of age) were obtained from the Animal Resources Centre, Western Australia, and maintained under specific-pathogen-free conditions for the studies. All animal experiments were approved by Animal Ethics Committee, Austin Health, Australia, and were carried out in accordance with the standards required by the Guidelines for the Welfare of Animals in Experimental Neoplasia (UKCCCR guidelines). The SCID mice were inoculated subcutaneously with CEM/A7R cells (2 × 10^7^). To examine antitumour efficacy of the Cripto Mab on an established tumour in SCID mice, treatment was not started until the tumours size had reached an average size of 100 mm^3^. C13 Mab (0.5 mg per mouse) was administrated intraperitoneally on day 6, then followed by 0.25 mg C13 twice a week for 3 weeks. Tumours were measured at 2–4 days intervals with a caliper, and tumour size was calculated (length × width × height) as described previously (Xing *et al*, 2004).

### Flow cytometry analysis

#### Annexin-V binding assay

Antibody-induced apoptosis was examined by Annexin V and propidium iodide (PI) dual staining to detect early apoptotic cells (Vermes *et al*, 1995). Cells (2 × 10^5^) were incubated with or without 25 *μ*g ml^−1^ of Cripto Mab for 4 h at 37°C in a humidified chamber containing 5% CO_2_. The Annexin binding assay was performed using the ApoTarget^Tm^ Annexin-V fluorescein isothiocyanate (FITC) Apoptosis kit (Biosource, CA, USA). The kit is designed to detect apoptotic cells by flow cytometry and examined with a FACScan flow cytometer (Becton Dickinson, Franklin Lakes, NJ, USA).

#### Cripto and Pgp expression

Cells (5 × 10^5^) were incubated with 10 *μ*g ml^−1^ of C13 or PE-conjugated UIC2 (Colter) at room temperature for 15 min. Nonspecific rat immunoglobulin (Ig)M and murine Mab IgG2a (Chemicon, CA, USA) were used as control for Cripto or Pgp expression, respectively. After three washes the cells were directly analysed for Pgp expression. For Cripto expression, the cells were further incubated with a fluorescein-conjugated sheep anti-rat antibody (Chemicon) at 1 : 80 dilution. Mean channel fluorescence (MCF) was recorded and the expression of Pgp, Cripto was shown as the ratios of the arithmetic MCF of UIC2 or C13 relative to the control antibodies (Hu *et al*, 1999a).

#### Rh123 accumulation

Rh123 is a fluorescence dye that can be taken up by the cells and pumped out by the Pgp pump. Rh123 accumulation in cells is a sensitive and selective measure of the transport function of Pgp, and can be detected by flow cytometry. Briefly, 5 × 10^5^ cells were incubated with or without 25 *μ*g ml^−1^ Cripto Mab C13, C4 or 10 *μ*m Vp for 3 h, respectively. The cells were washed and the fluorescence was measured after the addition of Rh123 at 200 ng ml^−1^ to the culture medium in the presence or absence of Mab or 10 *μ*m Vp. The cells were then incubated in the dark at 37°C for 1.5 h. Rh123 fluorescence was measured through a 530 DF 30-nm filter. The results were expressed as the ratios of MCF in the presence or absence of Vp or Mab (referred to as Rh123 ratio) (Hu *et al*, 1999a). An increase in Pgp function by Mab was defined as a change in the Rh123 ratio of treated and untreated cells.

### Western blot

Cells (5 × 10^6^) were treated with Mab C4 for various times in the presence or absence of AraC for 3 or 6 h. The cells were harvested, and washed with ice cold PBS, then lysed and sonicated in 0.5 ml lysis buffer containing 20 mM Tris (pH 7.5), 150 mM NaCl, 1 mM EDTA, 1 mM EGTA, 1% Triton X-100, 2.5 mM sodium pyrophosphate, 1 mM *β*-glycerolphosphate, 1 mM sodium orthovanadate (Na_3_VO_4_), 1 *μ*g ml^−1^ leupeptin and 1 mM phenylmethyl sulfonyl fluoride. The lysed samples were separated by 12.5 or 7.5% (for Pgp only) SDS-PAGE and transferred onto a polyvinylidene difluoride membrane (PVDF) (Amersham Pharmacia Biotech, Piscataway, NJ, USA). The blots were blocked with 5% no-fat dry milk in Tris-buffered saline buffer with 0.1% Tween-20 at room temperature for 1 h and probed with appropriate dilution of anti-Cripto Mab C13 or MDR1 (G-1) (Santa, Cruz, sc-13131), or cell signalling antibodies (New England Biolabs) including anti-phospho-Akt at Ser473 (#9271), anti-Phospho Bcl-2 at Ser 70 (#2871), anti-Bcl-XL (#2762), anti-Bad (#9292), anti-Phospho-Bad at Ser112 (# 9191), anti-Phospho-Bad at Ser136 (#9295) and anti-cleaved caspase 9 at Asp330 (#9501) antibodies. The proteins in the PVDF membranes were visualised using chemiluminescence reagent (Perkin-Elmer) after adding horseradish peroxidase-labelled secondary antibodies.

### JNK/SAPK assay

The JNK/SAPK activity was measured using JNK/SAPK assay kit (New England Biolabs) as described previously (Xing *et al*, 2004). Briefly, a recombinant fusion protein c-Jun residue 1–89 and glutathione *S*-transferase (GST-c-Jun) were used as the substrate for activated JNK. The supernatant of cell lysate was incubated with immobilised GST-c-Jun fusion protein overnight at 4°C to precipitate activated JNK. Kinase reaction was carried out *in vitro* at 30°C for 30 min in kinase reaction buffer (25 mM Tris–HCl (pH 7.5), 5 mM *β*-glycerolphosphate, 2 mM dithiothreitol, 0.1 mM sodium orthovanadate and 10 mM MgC1_2_) containing 100 *μ*M ATP. Phosphorylation of GST-c-Jun on Ser-63 was analysed by immunoblotting using anti-phospho-specific c-Jun (Ser-63).

### Statistical analyses

Two-way analysis of variance (ANOVA), GraphPad Prism Version 4.0 (GraphPad Software Inc., San Diego, CA, USA) was used to analyse the experiment results expressed as mean±s.d. The Mann–Whitney nonparametric *U*-test was used to compare the tumour sizes in groups of mice treated with Mab and PBS, respectively. *P*<0.05 was considered to be significantly different (Slinker, 1998).

## RESULTS

### Cripto expression and drug resistance

**Figure 1:**
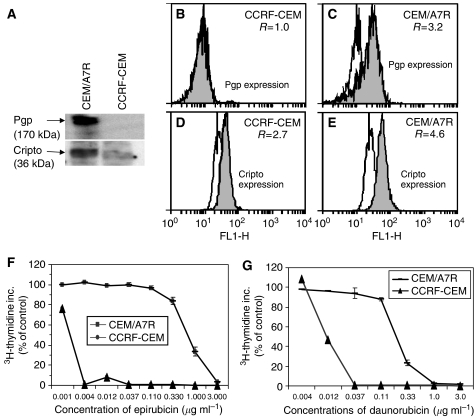
P-glycoprotein, Cripto expression and association with drug sensitivity in CEM/A7R and parental CCRF-CEM cells. (**A**) Western blot analysis of Cripto and Pgp expression in the CEM/A7R and CCRF-CEM cells using anti-Cripto Mab C13 and Mab to 1040–1280 amino acid of human Pgp. (**B** and **C**) P-glycoprotein expression measured by flow cytometric analysis using PE-conjugated UIC2 (solid histogram) compared to an IgG_2a_ (open histogram) and Pgp levels were expressed as the ratio of MCF of UIC2 *vs* a IgG_2a_ control in CCRF-CEM and CEM/A7R. (**D** and **E**) Cripto expression was measured by flow cytometry using C13 (solid histogram) compared to an IgM control (open histogram) in CCRF-CEM and CEM/A7R. Cripto levels were expressed as the ratio (R) of the MCF of C13 *vs* the IgM control. (**F** and **G**) Percentage of control in [^3^H]thymidine incorporation of CEM/A7R and CCRF-CEM in the presence of increasing concentrations of EPI and DAU for 48 h. Points are means of triplicate experiments. Error bars represent the s.d. in triplicate experiments.

To determine whether DOX selection of MDR CEM/A7R cells upregulated not only Pgp but also Cripto, Western blot analysis was performed using cell lysates of CEM/A7R and CCRF-CEM cells and probed by Mab to Pgp and anti-Cripto Mab C13. As shown in Figure 1A, MDR CEM/A7R cells overexpressed Pgp (170 kDa), whereas drug-sensitive cell line CCRF-CEM did not express Pgp. In contrast, though both cell lines expressed Cripto, CEM/A7R expressed higher level of Cripto than CCRF-CEM (Figure 1A). Flow cytometric analysis using PE-conjugated Pgp Mab UIC2 showed that ratios of MCF of UIC2 *vs* control IgG2a were 1.0 (8.8/8.9), 3.2 (34.5/10.7) in CCRF-CEM (Figure 1B) and CEM/A7R, (Figure 1C) respectively, implicating a threefold increase of Pgp expression in the CEM/A7R cells compared to parental CCRF-CEM cells. Cripto expression measured by C13 binding in flow cytometry analysis showed the ratios of Cripto expression were 2.7 (32.1/12.7) in CCRF-CEM (Figure 1D) and 4.6 (80.6/17.5) in CEM/A7R (Figure 1E) respectively, demonstrating 1.7-fold increase of Cripto expression in the CEM/A7R compared to the CCRF-CEM cells.

The Pgp-positive CEM/A7R cells were extremely resistant to EPI compared with the Pgp-negative CCRF-CEM cells. CEM/A7R cells showed 900-fold increase of resistance to EPI and 18.3-fold increase of resistance to DAU than its parental CCRF-CEM cells when compared at IC_50_ levels (0.9/0.001) for EPI (Figure 1F) and (0.22/0.012 of IC_50_s) for DAU (Figure 1G) in [^3^H]-thymidine incorporation assay, respectively.

### Inhibition of cell proliferation by Cripto Mab

**Figure 2:**
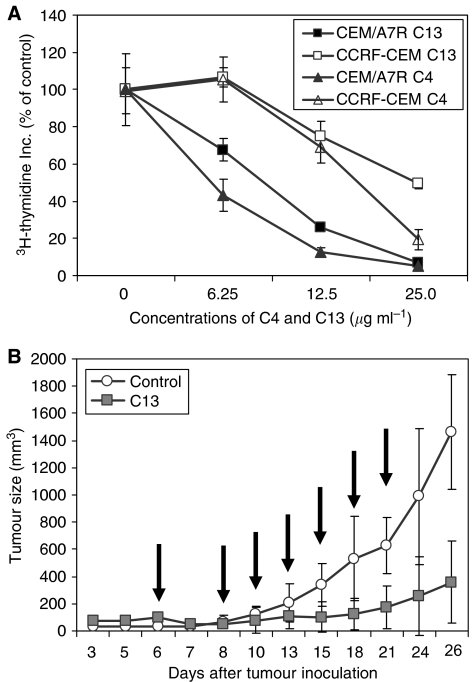
Inhibition of cell growth of CEM/A7R by anti-Cripto Mabs *in vitro* and *in vivo*. (**A**) Percentage of control in [^3^H]thymidine incorporation of CEM/A7R and CCRF-CEM in the presence of C4 and C13 for 48 h. Points are means of triplicate experiments. Error bars represent s.d. in triplicate experiments. (**B**) In *vivo* antitumour effect of anti-Cripto Mab C13 on established tumour of CEM/A7R xenografts in SCID mice. The SCID mice were inoculated s.c. with 2 × 10^7^ CEM/A7R MDR cells, and treated with 0.5 mg C13 on day 6 and 0.25 mg afterward (arrows) when the average size of the tumours was 100 mm^3^. Points show means and bars are s.d. of tumour size.

Anti-Cripto Mab C13 and C4 inhibited cell growth of both CEM/A7R and CCRF-CEM in a dose-dependent manner by the [^3^H]-thymidine incorporation assay. However, the MDR CEM/A7R cells were more sensitive to inhibition effects of C13 and C4 than CCRF-CEM cells. C13 at 6.25, 12.5 and 25 *μ*g ml^−1^ inhibited 32.3, 74.1 and 93.2% of [^3^H]-thymidine incorporation in CEM/A7R in contrast to 0, 25.2 and 50.7% of inhibition in CCRF-CEM, respectively (Figure 2A). The IC_50_ levels of C4 and C13 were 5.5 and 8.25 *μ*g ml^−1^ in the CEM/A7R cells and 18.8 and 25 *μ*g ml^−1^ in the CCRF-CEM cells. Therefore, compared to CCRF-CEM cells, CEM/A7R cells were three times more sensitive to antitumour activity of Cripto Mab (Figure 2A). This could be due to the difference in the levels of Cripto expression in the two cell lines (Figure 1A, D and E).

### Inhibition of MDR CEM/A7R tumour growth in SCID mice

The anti-MDR tumour effect of Cripto Mab was further investigated in MDR CEM/A7R xenograft model in SCID mice (Figure 2B). The SCID mice with established tumours (average size 101±17 mm^3^) were treated with C13 (0.5 mg per mouse) on day 6, followed by six injections of 0.25 mg (total of 2.0 mg per mouse) as indicated in Figure 2B. The tumour size was reduced significantly in the C13-treated group (300 mm^3^) compared with untreated control (1480 mm^3^, *n*=6; *P*<0.05), and 80% of reduced tumour size at day 26, 6 days after last C13 treatment, indicating the inhibitory effects of anti-Cripto Mab were durable, and may be of value in the treatment of drug resistant leukaemia (Figure 2B).

### Apoptosis induced by Cripto Mab

**Figure 3:**
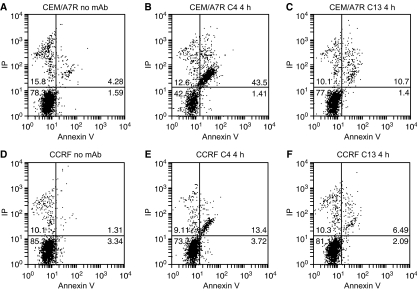
The percentage of dual staining of CEM/A7R and CCRF-CEM tested by flow cytometry using Annexin V and PI after treatment with control Mab BCP7 (anti-MUC1) (**A** and **D**) or 25 *μ*g ml^−1^ of anti-Cripto Mab C4 (**B** and **E**), C13 (**C** and **F**) for 4 h.

The binding of Annexin-V-FITC conjugates to cells permits differentiation of apoptotic cells from nonapoptotic cells. Flow cytometry analysis demonstrated 5.9, 44.9 and 12.1% of Annexin V-FITC and PI-double stained cells after 4 h incubation of CEM/A7R cells with medium (Figure 3A) only, C4 (Figure 3B) and C13 (Figure 3C). In contrast, there were 4.7% in medium (Figure 3D), 17.1% and 9.6% in C4 (Figure 3E) and C13 (Figure 3F) of Annexin V-FITC and PI-positive CCRF-CEM cells, respectively. The results indicated that the anti-Cripto Mab-induced higher proportions of apoptosis in CEM/A7R (Figure 3B and C) than CCRF-CEM cells (Figure 3E and F).

### Cripto Mab sensitise MDR cells to Pgp substrate EPI and DAU

**Figure 4:**
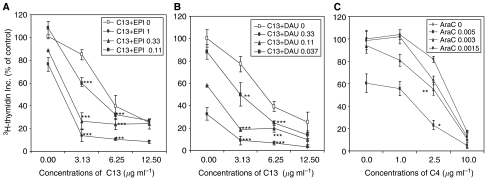
Inhibition of CEM/A7R cells by combined use of anti-Cripto Mab C13 with different concentrations of EPI at 0.11 to 1 *μ*g ml^−1^ (**A**), DAU at 0.04–0.33 *μ*g ml^−1^ (**B**) or C4 at various concentrations with Arac at 0.0015, 0.003 and 0.005 *μ*g ml^−1^ (**C**) after 48 h incubation as measured by percentage of control in [^3^H]thymidine incorporation. Points are means of triplicate experiments. Error bars represent s.d. in triplicate experiments. The interactions of various concentrations of C13 with EPI, DAU or C4 with AraC were subjected to two-way ANOVA. The *P-*values were marked as ^*^P<0.05, ^**^ *P*< 0.01, ^***^ *P*< 0.001.

To determine the effect of anti-Cripto Mab on enhancing cytotoxicity of EPI or DAU, a range of concentrations of each reagent was used on cell growth inhibition of CEM/A7R cells. Mab C13 inhibited 14.8, 60.2 and 74.7% of [^3^H]-thymidine incorporation at concentrations of 3.13, 6.25 and 12.50 *μ*g ml^−1^. As shown in Figure 4A, the addition of 3.1 *μ*g ml^−1^ of C13 to the culture containing noncytotoxic concentrations of 0.11, 0.33 and 1.0*μ*g ml^−1^ EPI inhibited 40.4, 73.5 and 86.2% of [^3^H]-thymidine incorporation. Combined use of 3.1 *μ*g ml^−1^ C13 with 0.037, 0.11 and 0.33 *μ*g ml^−1^ DAU showed growth inhibition of 50.2, 81.7 and 91.2%, respectively, compared to 11.8, 41.5, 67.5 inhibited by DAU alone (Figure 4B). Similar results were seen with 6.25 *μ*g ml^−1^ C13 in combination with EPI or DAU (Figure 4A and B). It is obvious that combined use of the anti-Cripto Mab and cytotoxic drugs significantly enhanced the inhibitory effect of each reagent compared with use alone. Importantly, more than 90% of [^3^H]-thymidine incorporation inhibition can be achieved by combined use of C13 at less than 50% of inhibition concentrations with noncytotoxic concentrations of EPI or DAU (Figure 4A and B). Two-way ANOVA analysis demonstrated that there were synergistic effects in most combinations of C13 and EPI or DAU (*P*<0.05 or *P*<0.01, *P*<0.001 as indicated by ^*^, ^**^, ^***^ in Figure 4A and B). These results indicate that Cripto Mab C13 at less than IC_50_ concentrations could reverse drug resistance in the MDR CEM/A7R cells. Similar results were observed in the combined use of C4 and EPI or DAU (data not shown).

### Cripto Mab sensitise MDR cells to non-Pgp substrate AraC

Non-Pgp substrate AraC at noncytotoxic concentrations of 0.0015, 0.003 and 0.005 *μ*g ml^−1^ (⩽IC_50_) generated 1.4, 5.7 and 39.7% of inhibition of [^3^H]-thymidine incorporation respectively in CEM/A7R cells. C4 alone inhibited 0, 18.2 and 82.9% of [^3^H]-thymidine incorporation of CEM/A7R at concentrations of 1.0, 2.5 and 10.0 *μ*g ml^−1^, respectively. The addition of 2.5 *μ*g ml^−1^ C4 to the tissue culture medium containing 0.0015, 0.003 and 0.005 *μ*g ml^−1^ of AraC generated 37.1, 44.7 and 76.9% of inhibition of [^3^H]-thymidine incorporation, respectively, in the CEM/A7R cells. A 95.2% of inhibition was obtained by combined use of 10.0 *μ*g ml^−1^ C4 and 0.005 *μ*g ml^−1^ AraC (Figure 4C). The results indicated that combined use of the anti-Cripto Mab and AraC significantly enhanced the inhibitory effect than when they were used alone. Two-way ANOVA analysis demonstrated the combined use of C4 and AraC had a synergistic effects (*P*<0.05 or *P*<0.01, as indicated by ^*^, or ^**^ in Figure 4C). A synergistic effect was also observed in combination of C13 or C4 and AraC in CCRF-CEM cells (data not shown).

### Rh123 accumulation by Cripto Mab

**Figure 5:**
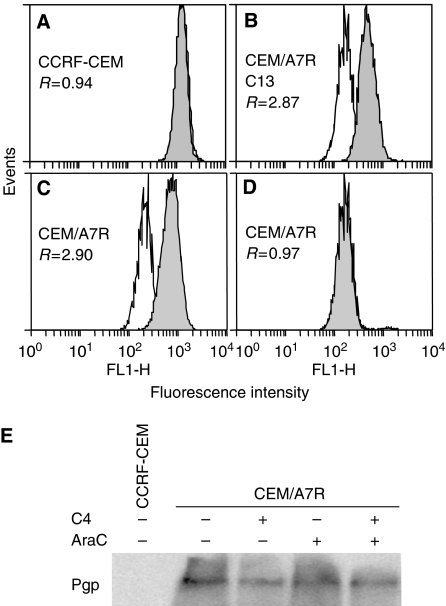
P-glycoprotein function and expression affected by anti-Cripto Mab in CCRF-CEM and CEM-A7R tested by flow cytometry and Western blot. (**A** and **C**) Flow cytometry analysis of Rh123 accumulation in the absence (open histogram) or presence of 10 *μ*M Vp (solid histogram). (**B**) Rh123 accumulation in the CEM/A7R cells following 3 h treatment with 25 *μ*g ml^−1^ C13 in the absence (open histogram) or presence of 10 *μ*M Vp (solid histogram) or in the absence (open histogram) or presence of 25 *μ*g ml^−1^ C13 (solid histogram) (**D**). P-glycoprotein function is expressed as the ratio of MCF in the presence or absence of Vp or C13 as described in the ‘Materials and Methods’. P-glycoprotein levels were expressed as the ratio (R) of the MCF. (**E**) Western blot analysis of Pgp expression detected by Mab to human Pgp in the CEM/A7R cells following 6 h treatment with 10 *μ*g ml^−1^ C4, 0.02 *μ*g ml^−1^ AraC and combined use of the two reagents.

The effects of C13 on Pgp function were determined by comparison with Pgp modulator Vp on intracellular accumulation of Rh123 fluorescence, a sensitive assay for assessment of Pgp function and the results were expressed as ratios of MCF of Rh123 (Hu *et al*, 1999a). P-glycoprotein modulator Vp at 10 *μ*M increased Rh123 accumulation in CEM/A7R cells by 2.9-fold (751/259) (Figure 5C) and had no significant effect on the Pgp-negative CCRF-CEM cells (ration 0.94; 1275/1361) (Figure 5A). The data demonstrated that overexpression of Pgp resulted in threefold decrease (2.90/0.90) of Rh123 accumulation in CEM/A7R than CCRF-CEM cells (Figure 1A and C). Figure 5B and 5D shows the changes of Rh123 accumulation in the CEM/A7R cells following 3 h incubation with C13. Verapamil increased accumulation of Rh123 at similar extent (ratio 2.87 : 526/183) (Figure 5B) compared to the CEM/A7R cells (ratio 2.90) (Figure 5C). In contrast, C13 alone had little effect on intracellular Rh123 in the CEM/A7R cells after C13 treatment (ratio 0.97 : 181/186) (Figures 5D). Similar results were seen in the cells treated with C4 (data not shown). The results indicated that C13 and C4 had little effect on inhibition of Pgp function, suggesting the effects of anti-Cripto Mab on the overcoming drug resistance were Pgp independent.

### The effect of Cripto Mab and AraC on regulation of Pgp expression

It has been previously shown that Pgp substrates anthracyclines and its analogues as well as non-Pgp substrate AraC differentially upregulated Pgp expression in the CEM/A7R cells (Hu *et al*, 1995, 1999b) and in blast cells from AML, respectively (Hu *et al*, 1999a). In the present study, the regulation of Pgp expression by C4 or AraC and the combined use of C4 and AraC were analysed by Western blot and flow cytometry. CEM/A7R cells were treated for 6 h with 10 *μ*g ml^−1^ C4, 0.02 *μ*g ml^−1^ AraC individually or in combination. Western blot analysis showed C4 slightly decreased Pgp expression in CEM/A7R cells in the presence or absence of AraC (Figure 5E). P-glycoprotein expression had no significant changes in ratios tested by flow cytometry following the above treatment (data not shown).

### Activation of SAPK/JNK signalling pathway

**Figure 6:**
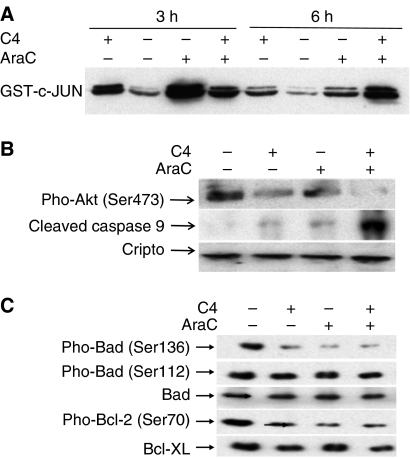
Western blot showing signalling molecules mediated by C4, AraC and combination of the two reagents in the CEM/A7R cells. (**A**) Activation of JNK/SAPK examined by JNK kinase assay and blotted by anti- phosphor-c-Jun Mab in CEM/A7R cells treated with 10 *μ*g ml^−1^ C4 and 0.02 *μ*g ml^−1^ AraC or combination for 3 or 6 h. (**B**) Western blot analysis of phospho-Akt at Ser473 and cleavage of caspase 9 at Asp330 compared to the level of expression of Cripto and (**C**) Western blot analysis of phosphor-Bad at Ser136, Ser112, total Bad protein, phosphor-Bcl-2 at Ser70, Bcl-xL in the same cell lysates.

Signalling events involved in the anti-MDR tumour effects of Cripto Mab on MDR CEM/A7R cells were examined by comparison of C4 and non-Pgp substrate AraC and their combination on the activation of stress-activated protein kinase/c-Jun N-terminal kinase (SAPK/JNK) proapoptotic pathways. Stress-activated protein kinase/c-Jun N-terminal kinase is activated by multiple forms of stress including UV, radiation, inflammatory cytokines, and has been implicated as a mediator of stress-induced apoptosis (Davis, 2000). A sustained activation of SAPK/JNK led to cancer cell apoptosis (Xia *et al*, 1995). Activation of SAPK/JNK proapoptotic pathways was examined by using JNK kinase assay in the cell lysates from CEM/A7R cells treated with 10 *μ*g ml^−1^ C4, 0.02 *μ*g ml^−1^ AraC for 3 and 6 h or in combination (Figure 6A). c-Jun N-terminal kinase activity was increased at 3 h of incubation of CEM/A7R cells with 10 *μ*g ml^−1^ C4 compared to control, and remained at the elevated level by 6 h. In contrast, 0.02 *μ*g ml^−1^ AraC significantly activated JNK with higher level than C4 at 3 h incubation, and declined at 6 h. Combined use of C4 and AraC-enhanced JNK activity compared to C4 and AraC alone by 6 h (Figure 6A). The results indicated that C4 and AraC alone activated JNK activity and combination treatment showed a sustained enhancement of JNK activity by 3 and 6 h (Figure 5A).

### Inhibition of Akt by Cripto Mab

Tumorigenesis of Cripto is related to its activation of c-Src, MAPK and PI3K/Akt pathways (Kannan *et al*, 1997; Ebert *et al*, 1999; Bianco *et al*, 2003). Akt is activated by Cripto through phosphorylation of Ser473 at the COOH-terminus to promote cell survival and proliferation (Ebert *et al*, 1999). Moreover, JNK-dependent apoptotic signalling pathway can be blocked by activation of survival signalling Akt (Xia *et al*, 1995). An inhibition of Phospho-Akt at Ser473 has been demonstrated in Western blot analysis of CEM/A7R cells after 3 h incubation with 10 *μ*g ml^−1^ C4 or 0.02 *μ*g ml^−1^ AraC, respectively (Figure 6B). The most effective inhibition of phospho-Akt was observed by incubation of CEM/A7R with 10 *μ*g ml^−1^ C4 and 0.02 *μ*g ml^−1^ AraC (Figure 6B). No changes in the levels of P44/42 (MAPK) or p38 were shown after treatment of the cells with C4 or AraC for 3 h (data not shown). The expression of Cripto was unchanged before and after treatment (Figure 6B).

### Caspase-9 cleavage

Caspase 9 is an initiator for caspase. Cleaved caspase 9 is an activated form of caspase 9 and further processes other caspases, including caspase 3 and caspase 7, which amplify the cascade leading to apoptosis. c-Jun N-terminal kinase is required for the stress-induced apoptosis mediated by mitochondrial/caspase-9 pathway (Tournier *et al*, 2000). Akt phosphorylates caspase-9 and inhibits caspase-9 protease. As shown in Figure 6A and B, JNK activation and Akt inhibition by C4 and AraC were associated with the appearance of cleaved caspase-9 at Asp330 (37 kDa). The most enhanced induction of cleaved caspase-9 was induced by combined use of C4 and AraC (Figure 6B). This may explain the synergistic effect of anti-Cripto Mab to sensitise MDR CEM/A7R cells to AraC.

### Bad, Bcl-2 as apoptosis targets of anti-Cripto Mab

Bad is a proapoptotic member of the Bcl-2 family that is capable of forming heterodimers with Bcl-2 or Bcl-xL, and antagonises their antiapoptotic activity leading to cell death. Phosphorylation of Bad at Ser 136 by Akt or at Ser 112 through Ras/Raf/MAPK cascades resulted in the binding of Bad to 14-3-3 proteins, and inhibition of Bad binding to Bcl-2, Bcl-xL in promotion of cell survival (Zha *et al*, 1996). Thus, only dephosphorylation of Bad is associated with apoptosis. In the CEM/A7R cells, we demonstrated that C4 at 10 *μ*g ml^−1^ or AraC at 0.02 *μ*g ml^−1^ dramatically decreased phosphorylation of Bad at Ser136, but not at Ser112. C4 and AraC also significantly inhibited phosphorylation of Bcl-2 at Ser70, which was associated with cell survival in CEM/A7R. Treatment of CEM/A7R cells with combined use of C4 and AraC further decreased phosphorylation of Bad at Ser136, Bcl-2 at Ser70 and slightly decreased Bcl-xL. However, total Bad protein was not affected by any treatment (Figure 6C).

## DISCUSSION

Multidrug resistance related protein 1 (MDR1) related multiple-drug resistance remains a major impediment undermining successful cancer treatment. Despite the recent developments of understanding the mechanisms, there is no effective method to treat relapsed cancer, which has developed MDR after failure of chemotherapy. Many studies focus on overcoming MDR through modulation of Pgp function. These include Pgp modulators Vp, CyA, PSC833, and recently developed anti-CD19 (Ghetie *et al*, 1999), anti-CD20 Mab (Demidem *et al*, 1997) and anti-Pgp Mab (Smyth *et al*, 1998). In addition to inhibition of Pgp function as drug efflux pump, these agents also showed inhibition of MDR1 expression during chemotherapy (Hu *et al*, 1996, 1999a) or interrupted interactions between Pgp and targeted antigen by antibodies leading to a dysfunction of Pgp (Ghetie *et al*, 2004). However, Pgp not only functions as a drug efflux pump, but also interrupts apoptotic pathways (Smyth *et al*, 1998; Johnstone *et al*, 1999). Here we demonstrated a distinguished strategy to overcome MDR in CEM/A7R cells by induction of cancer cell apoptosis signalling pathways using Mab to Cripto, a recently identified unique target for cancer therapy (Adkins *et al*, 2003; Xing *et al*, 2004; Hu and Xing, 2005). Cripto was found to be overexpressed in the CEM/A7R cells that acquired Pgp-mediated MDR by DOX treatment (Zalcberg *et al*, 1994). Activation of c-Src and MAPK/PI3K/Akt pathways (Bianco *et al*, 2003) by Cripto involves in the cell proliferation and survival, resulting in an intervention of chemotherapeutics-induced apoptosis pathways and promotes MDR as demonstrated in several cancers (Grandage *et al*, 2005). Furthermore, activation of PI3K/Akt pathway has been suggested in controlling MDR mediated by Pgp and the MRP1, MRP2 (Abdul-Ghani *et al*, 2006). As MDR1 and Cripto are *β*-catenin target genes (Yamada *et al*, 2000; Morkel *et al*, 2003), coexpression of Pgp and Cripto in CEM/A7R cells could be simultaneously induced by intracellular accumulation of *β*-catenin during DOX selection, resulting in multiple defects in apoptosis pathways (Watts *et al*, 2001). We demonstrated that Mab to a 17-mer peptide within the EGF-like region of Cripto, C4 for example, induced massive induction of apoptosis in the CEM/A7R cells (Figure 3), which is three times higher than that in the Pgp-negative CCRF-CEM cells, and correlated to the expression levels of Cripto (Figure 1A and E). As a result, these anti-Cripto Mab inhibited Pgp-positive CEM/A7R cell growth *in vitro* and established tumour growth *in vivo* (Figure 2).

Molecules known to predispose cells to apoptosis have shown to enhance sensitivity of tumour cells to a variety of chemotherapeutic agents (Fisher, 1994). We propose that anti-Cripto Mab could overcome MDR phenotype in Pgp expressing MDR cells by induction of apoptosis. As expected, anti-Cripto Mab overcame MDR, and combined use of Cripto Mab C13 with anthracyclines completely reversed resistance of MDR CEM/A7R cells to EPI and DAU (Figure 4A and B). These observations indicated that the residual of the drug-resistant tumour cells could be eradicated by the addition of low concentrations of anti-Cripto Mab to the originally unresponsive concentrations of chemotherapeutic Pgp substrates to prevent tumour cells from recurrence. Synergistic effect was also observed between interactions of anti-Cripto Mab and non-Pgp substrate AraC (Figure 4C). The findings may be clinically significant, because AraC has been used for many years in the treatment of AML, and the resistance to AraC remains a major obstacle in the effective treatment (Fernandez-Calotti *et al*, 2005). We also demonstrated that the reversal of Pgp-mediated MDR by anti-Cripto Mab in the Pgp-positive CEM/A7R cells is irrelevant to Pgp function (Figure 5). These results indicated that MDR could be targeted by anti-Cripto Mab to bypass the Pgp through targeting a signal molecule to induce apoptosis in MDR tumour cells with overexpression of Cripto and Pgp. This strategy could be superior to targeting Pgp function as a low level of Pgp is expressed in many normal tissue cells such as liver, biliary tract, brain, kidney and intestines, which play roles in the excretion of toxins. Modulation of Pgp function caused decreased excretion of drugs and enhanced toxicities, such as nausea and vomiting, increased myelosuppression and hyperbilirubinemia (Szakacs *et al*, 2006). No obvious side effect was observed in the mouse model treated by anti-Cripto Mab. However, it should be investigated in future clinical trials using humanised Mab.

The molecular basis for anti-Cripto Mab in the induction of apoptosis and enhancing cytotoxicity of chemotherapeutics was examined on activation of JNK/SAPK and inhibition of Akt pathways. We found that JNK/SAPK pathway was activated by anti-Cripto Mab and AraC in the CEM/A7R cells. Combined use of Mab and AraC enhanced JNK activation by 6 h compared with them used alone (Figure 6A). Stress-activated JNK/SAPK apoptosis pathway is closely related to an alteration of dual function of Axin, a multidomain scaffold protein, which coordinates a variety of critical factors in determination of activation of JNK-apoptotic pathway or Wnt oncogenic signalling pathway (Zhang *et al*, 2001). In the CEM/A7R cells, PI3K/Akt pathway is intrinsically activated by Cripto (Ebert *et al*, 1999). Presumably, anti-Cripto Mab inhibits phosphorylation of Akt, a major molecule of PI3K/Akt pathway and interrupted Cripto/Akt/GSK-3*β* signalling pathways leading to destabilising *β*-catenin (Neo *et al*, 2000) and switching Axin to activate JNK apoptosis signalling instead of favouring in Wnt signalling activation (Zhang *et al*, 2001). Indeed, the level of Akt phosphorylation at Ser473 was reduced by the anti-Cripto Mab, indicating the Mab acted as an inhibitor of Akt activation (Figure 6B). Targeting Akt pathway has been successful in attenuating chemotherapeutic resistance by small molecule inhibitor LY294002 (West *et al*, 2002). However, side effects and acquired resistance have limited in clinical use of these small molecular inhibitors. Moreover, many forms of cellular stress including chemotherapeutic cytotoxics can also upregulate PI3K/Akt signalling (West *et al*, 2002). Stress-induced activation of PI3K/Akt pathway is regarded as a protective compensatory mechanism for cells to escape chemotherapy-induced cell death (Martelli *et al*, 2006). A rapid upregulation of PI3K/Akt pathway has been observed in DAU-treated U937 human leukaemia cells (Plo *et al*, 1999). In contrast to DAU, AraC inhibited phosphorylation of Akt at Ser473, which was further abolished by combined use of C4 with AraC (Figure 6B). Therefore, the anti-Cripto Mab may be promising as a nonsmall molecule inhibitor in clinical use to inhibit activation of Akt signal pathway during chemotherapy.

Two principal pathways are involved in apoptosis: a pathway that is directly activated by death receptors and a pathway that involves mitochondria. Studies on the role of JNK in apoptotic signalling revealed that JNK is required for the stress induced apoptosis mediated by mitochondrial/caspase-9 pathway (Tournier *et al*, 2000). Caspase-9, an important molecule to mediate mitochondrial pathway, is a cell death protease, which is phosphorylated and inactivated by Akt, and is cleaved by the cytochrome *c* to initiate caspase cascade with requirement of JNK/SAPK (Tournier *et al*, 2000). Our results showed that Cripto Mab and AraC-induced cleavage of caspase 9 at Asp 330, which were greatly amplified by the combined treatment of C4 and AraC, indicating the activation of mitochondria/caspase-9 apoptosis pathway is associated with a synergistic increase in the induction of apoptosis (Figures 4C and 6B). The combination of C4 and AraC had greater effects on JNK/SAPK activation and inhibition of Akt phosphorylation that correlates a synergistic increase in the induction of cleaved levels of casepase 9 (Figure 6A and B).

The mitochondria apoptosis pathway is primarily regulated by Bcl-2 family proteins consisting of anti-apoptotic Bcl-2 and Bcl-xL and proapoptotic members Bad and Bax (Petros *et al*, 2004). Bad forms a heterodimer with the anti-apoptotic proteins Bcl-2 or Bcl-xL and thereby prevents them from exerting their antiapoptotic function. Several signalling pathways influence cell death through their effects on the phosphorylation of Bad at various sites (Downward, 1999). Akt phosphorylates Bad at Ser136, and promotes the association of Bad with 14-3-3 proteins in the cytosol and inactivates Bad pro-apoptotic function (Datta *et al*, 1997). Only the nonphosphorylated Bad heterodimerised with Bcl-xL at mitochondria membrane sites to promote cell death. c-Jun N-terminal kinase/stress-activated protein kinase phosphorylated Bcl-2 at Ser70 *in vitro* and *in vivo*. Phosphorylation of Bcl-2 at Ser70 inhibits the antiapoptotic function of Bcl-2 (Yamamoto *et al*, 1999). Anti-Cripto Mab, AraC dramatically decrease phosphorylation of Bad at Ser136 and phosphorylation of Bcl-2 at Ser70 in the CEM/A7R cells, and had little effect on phosphorylation of Bad at Ser112, total level of Bad and Bcl-xL. The combined use of Mab and AraC induced further decrease of phosphorylation of Bad at Ser136 and Bcl-2 at Ser70 in the CEM/A7R cells (Figure 6C). The data demonstrate that a mitochondrial cell death pathway induced by anti-Cripto Mab is involved in the inhibition of cell growth and overcoming drug resistance in the MDR CEM/A7R.
